# Myxedema Coma Precipitated by Sepsis in a Patient With a Complex Mental Health History

**DOI:** 10.7759/cureus.43574

**Published:** 2023-08-16

**Authors:** Mehul S Amin, Umesh Kumar Pabani, Sunaina Lohano, Zahid Khan

**Affiliations:** 1 Internal Medicine, Southend University Hospital, London, GBR; 2 Internal Medicine, Mid and South Essex NHS Foundation Trust, Southend-on-Sea, GBR; 3 Geriatrics, Newham University Hospital, London, GBR; 4 Acute Medicine, Mid and South Essex NHS Foundation Trust, Southend-on-Sea, GBR; 5 Cardiology, Bart’s Heart Centre UK, London, GBR; 6 Cardiology and General Medicine, Barking, Havering and Redbridge University Hospitals NHS Trust, London, GBR; 7 Cardiology, Royal Free Hospital, London, GBR

**Keywords:** mental health comorbidities, thyroid stimulating hormone tsh, severe sepsis, myxedema crisis, hypothyroid myxedema coma

## Abstract

Myxedema coma is a rare and life-threatening manifestation of severe hypothyroidism. Myxedema refers to altered mental status observed in these patients. Clinical characteristics observed include hypothermia, bradycardia, respiratory failure, hyponatremia, and altered mental status. We present the case of a 57-year-old female who was brought into the hospital with a history of collapse and a long lie. On initial assessment, she was hypothermic, hypotensive, bradycardic, and hypoglycemic with elevated infection markers, acute kidney injury, and electrolyte derangement. Her thyroid function tests on admission were severely impaired with a TSH (thyroid stimulating hormone) level of 144.46 mU/L and Free T4 (thyroxine) levels of 3.4 pmol/L. She was admitted to the intensive care unit and was started on intravenous antibiotics, intravenous liothyronine, oral levothyroxine, and intravenous hydrocortisone. Initially, her hypothermia and bradycardia were slow to respond to treatment measures, but following the introduction of liothyronine, she showed marked improvement. Over the next few days, her infection markers improved, her acute kidney injury resolved, and her thyroid function tests normalized. Liothyronine was stopped after 6 days, levothyroxine was continued at her regular dose of 175 micrograms, and she was safely discharged with outpatient endocrinology follow-up.

## Introduction

Myxedema coma is a rare and life-threatening condition that can present with altered mental status, hypothermia, bradycardia, respiratory failure, and hyponatremia [1-3]. The development of myxedema coma occurs secondary to precipitating factors such as poor medication compliance, post-thyroidectomy, acute events (including infection, and myocardial infection amongst other stressors), and medication leading to loss of thyroid homeostasis [2,4]. The development of myxedema coma occurs secondary to precipitating factors, with the two most common causes of hypothyroidism being autoimmune thyroiditis worldwide and iodine deficiency in the United States [5,6]. Precipitants include poor medication compliance, post-thyroidectomy status, acute events such as infections, myocardial infarction, and medications disrupting thyroid homeostasis [2,4].

The recognition of myxedema often is difficult and complex. It is therefore vital to have a high degree of suspicion to recognize and commence prompt treatment due to high mortality. This may be complemented by a thorough history comprising thyroid surgery, medication history, and compliance with medication. Admission to the intensive care unit (ICU) should be considered as part of the management for monitoring and organ support. The mainstay of treatment includes hydrocortisone, intravenous liothyronine, levothyroxine, electrolyte correction, supportive therapies, and treating the underlying precipitant such as infection, surgery, or trauma [3,4]. Most patients with myxedema coma present with altered main status and it is vital to recognize this important feature of the disease presentation [5]. Patients suspected to have myxedema coma should receive intravenous hydrocortisone while awaiting laboratory results as delay can be life-threatening. Myxedema coma patients should ideally be managed in ICU with pulmonary and cardiovascular support [5,7]. Herein we present a case of a female hypothyroid patient who presented with a history of a collapse and a long lie with multiorgan dysfunction as a result of decompensation of the thyroid gland.

## Case presentation

We present the case of a 57-year-old female who was brought into the emergency department (ED) as a trauma call with a history of collapse and a long lie at home. She lived alone, normally independent, and was found on the floor by a neighbor who last saw her three days prior. The ambulance crew found that she was less responsive with a Glasgow Coma Scale (GCS) of 10/15 (E4V1M5), and evidence of vomiting along with pressure sores on her torso and injury to her face. Vitals showed a respiratory rate of 10 breaths per minute and unrecordable oxygen saturation on pulse oximetry, therefore, she was started on 15 L/min oxygen via a non-rebreathing mask; blood pressure was unrecordable and her pulse rate was 36 beats per minute on cardiac monitoring. Her capillary blood glucose was 3.8 mmol/L. On initial clinical assessment, she was maintaining her airway and was cold to the touch. Chest examination revealed bibasal fine crepitation and sinus bradycardia with a heart rate of 36 bpm. There was evidence of injury marks on her face, chest, and both knees; her abdomen was soft and non-tender, and her calf muscles were soft, non-tender, and not swollen to suggest deep vein thrombosis. There was no rash identified on clinical examination. Her neurological assessment was normal apart from low GCS. She was commenced on warm intravenous (IV) normal saline 0.9% as her temperature was unrecordable during observations, and passive warming with warm blankets was also commenced.

Her past medical history (PMH) was significant for hypothyroidism (diagnosed in 2014) anxiety, depression, paranoid schizophrenia (diagnosed in 2021), and hypertension. She normally took mirtazapine 15 mg once at night, risperidone 3 mg once in the morning, ramipril 10 mg once in the morning, levothyroxine 175 mcg once in the morning, and felodipine 2.5 mg once in the morning. She did not have any medication compliance issues and there was no evidence of drug overdose or intoxication based on history and clinical examination supported by lab results.

**Figure 1:**
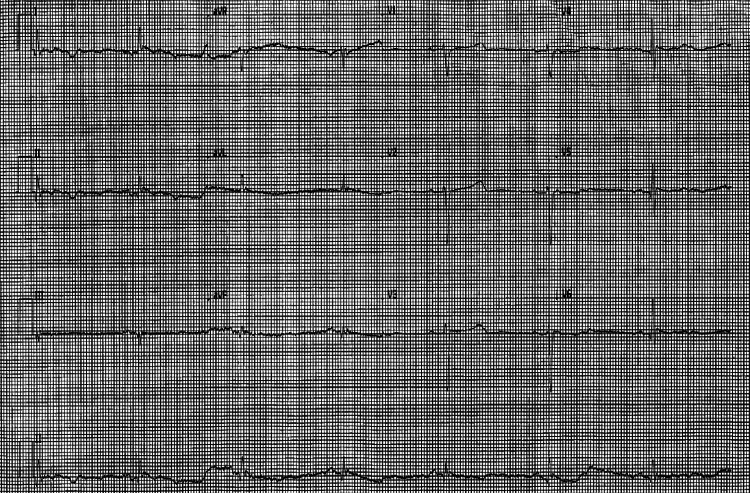
Electrocardiogram showing sinus bradycardia on admission

She had a CT (computed tomography) trauma scan of the head, cervical spine, thorax, abdomen, and pelvis that demonstrated congestion in the lower zones of both lungs but no obvious fracture or intracranial bleed was identified. Her electrocardiogram (ECG) showed sinus bradycardia with a heart rate of 36 bpm and a corrected QT interval of 461 milliseconds (Figure 1). The arterial blood gas (ABG) on 85% FiO2 (fraction of inspired oxygen) showed metabolic acidosis with partial respiratory compensation and no evidence of respiratory failure (Table 1). She was put on 2 liters of oxygen via nasal cannula following her ABG. She was started on dextrose 10% for low blood sugar. Her admission blood tests showed raised inflammatory markers, multiple electrolyte abnormalities, and acute kidney injury stage 3 (Table 2).

**Table 1:** Arterial blood gas on admission pCO_2_: partial pressure of cabon dioxide; pO_2_: partial pressure of oxygen; HCO_3_: bicarbonate

Parameter	Value	Normal Range
pH	7.31	7.35 - 7.45
pCO_2_	3.13 Kpa	4.67 - 6.00 KPa
pO_2_	70.1 Kpa	10.7 - 13.3 KP
HCO_3_	14.8 mmol/L	22 – 26 mmol/L
Base Excess	-14.4 mmol/L	-2 - +2 mmol/L
Lactate	0.8 mmol/L	0.4 - 2.2 mmol/L
Glucose	3.8 mmol/L	4.4 - 6.1 mmol/L

**Table 2:** Laboratory blood tests values during admission

Blood Test	Value Day 1	Value Day 3	Value Day 7	Normal Range
Hemoglobin	112 g/L	100 g/L	102 g/L	133 - 173 g/L
White cell count (WCC)	1.9 * 10^9^/L	4.8 * 10^9^/L	8.1 * 10^9^/L	3.8 - 11 * 10^9^/L
Platelet count	223 * 10^9^/L	127 * 10^9^/L	163 * 10^9^/L	150 – 400 * 10^9^/L
C reactive Protein (CRP)	229 mg/L	119 mg/L	8 mg/L	0 - 5 mg/L
Prothrombin time (PT)	12.9 seconds	10.7 seconds	11.9 seconds	10.3 - 13.3 seconds
Activated partial thromboplastin time	28.8 seconds	25.7 seconds	22.5 seconds	25.7 - 35.3 seconds
Amylase	44 U/L	142 U/L	103 U/L	28 – 100 U/L
Creatine kinase (CK)	1062 U/L	-	-	<200 U/L
Sodium (Na)	123 mmol/L	145 mmol/L	145 mmol/L	133 - 146 mmol/L
Potassium (K)	3.2 mmol/L	4.8 mmol/L	4.4 mmol/L	3.5 - 5.3 mmol/L
Urea	25.8 mmol/L	16.1mmol/L	14.3 mmol/L	2.5 -7.8 mmol/L
Creatinine	323 umol/L	104 umol/L	77 umol/L	59 - 104 umol/L
Thyroid Stimulating Hormone (TSH)	144.46 mU/L	30.67 mU/L	3.04 mU/L	0.3 - 5.0 mU/L
Free T4 (thyroxine)	3.4 pmol/L	9.3 pmol/L	12.6 pmol/L	7.9 - 16.0 pmol/L
Albumin	23 g/L	22 g/L	25 g/L	35 – 50 g/L
Corrected calcium	1.89 mmol/L	2.21 mmol/L	2.20 mmol/L	2.20 - 2.60 mmol/L
Cortisol (random)	>1655 nmol/L	-	-	185 - 624 nmol/L

As part of ongoing management, she was catheterized, sepsis screening was done and she was started on co-amoxiclav 1.2 grams intravenously twice a day; 10 milliliters of calcium gluconate 10% was given to correct the calcium level; and she was admitted to the intensive care unit for ongoing monitoring and management. A couple of hours after being in the intensive care unit, her first recordable temperature was 29.3°C. She was treated with active and passive measures including warm intravenous fluids and blankets. After 12 hours, her temperature was still 33°C, and her heart rate was 56 per minute. Due to such a high TSH value, and presentation of hypothermia, hypotension, hypoglycemia, and bradycardia, she was discussed with the on-call Endocrinologist regarding the probability of myxedema coma as a likely contributing factor along with sepsis for the slow recovery and response to treatment. Cortisol level is usually high during acute severe illnesses in patients without hypothalamic-pituitary-adrenal disease.

Her myxedema coma score was 155 (Table 3).

**Table 3:** MCS and patient's total score MCS: Myxedema Coma Score; GFR: glomerular filtration rate * Highlighting the patient's scores for different categories. ** Other EKG (electrocardiogram) changes include bradycardia, low QRS voltages, and widespread T wave inversions.

Thermoregulatory dysfunction (Temperature °F/°C)	Points
>95/35	0
89.6-95/32-35	10
<89.6/32	20 *
Central Nervous System Effects	
Absent	0
Somnolent/Lethargy	10
Obtunded	15
Stupor	20
Coma/seizures	30 *
Gastrointestinal Findings	
Anorexia/abdominal pain/constipation	5 *
Decreased intestinal motility	15
Paralytic ileus	20
Precipitating Event	
Absent	0
Present	10 *
Cardiovascular Dysfunction	
Bradycardia/Heart rate	
Absent	0
50-59	10
40-49	20
<40	30 *
Other EKG changes**	10
Pericardial/pleural effusion	10
Pulmonary edema	15
Cardiomegaly	15
Hypotension	20 *
Metabolic Disturbances	
Hyponatremia	10 *
Hypoglycemia	10 *
Hypoxemia	10 *
Hypercarbia	10
Decrease in GFR	10 *
Total Score	155

She was commenced on intravenous liothyronine 20 micrograms twice a day and intravenous hydrocortisone 50 milligrams four times a day by the Endocrinologist. Her regular levothyroxine of 175 micrograms was continued and administered through a nasogastric tube. Subsequently, in the following 12 hours, her temperature improved to 36.4°C, her heart rate increased to 87 per minute and her hypotension also resolved. 72 hours (about 3 days) post-treatment with liothyronine, her thyroid function tests showed improvement. Liothyronine was stopped after 6 days and thyroid function tests on day 7 showed normal thyroid function as shown in Table 2. Following this, only oral levothyroxine was continued at a dose of 175 micrograms once a day. Her blood cultures did not show any growth and therefore antibiotics were discontinued after completing a five-day course. She remained stable thereafter and was successfully discharged from the hospital with an outpatient Endocrinology review. She had a telephonic appointment about a month later and remained stable.

## Discussion

Our patient had multiorgan dysfunction comprising altered mental status, cardiovascular collapse, renal impairment, and electrolyte derangement, and upon recognizing the features of severe hypothyroidism, the patient required ICU admission for thyroid hormone replacement and intensive monitoring due to a combination of multiorgan dysfunction and sepsis possibly secondary to a chest infection. Renal functions can happen in patients with long lie due to rhabdomyolysis and dehydration resulting in raised creatinine kinase but also myopathy secondary to hypothyroidism [7,8]. Therefore, simply providing intravenous hydration alone would not be sufficient to reverse the renal impairment and thus requires optimizing thyroid function.

The clinical features observed in the above case could also mimic other conditions including sepsis and adrenal insufficiency would present with multiorgan dysfunction, altered mental status, and electrolyte derangement. In adrenal insufficiency biochemical markers show metabolic acidosis, hyperkaliemia, hyponatremia, and hypoglycemia. The administration of glucocorticoids first before other treatments is vital due to hypoadrenalism being a major risk factor for mortality and may co-exist with severe hypothyroidism [9]. In the above patient, however, cortisol was within normal range. The above case highlights the importance of early recognition and escalation of suspected myxedema coma to intensive care in known cases of hypothyroidism. The use of a myxedema coma diagnostic tool such as that developed by Popovenuic et al can help guide diagnosis in suspected cases which requires a score of 60 or greater [10]. Thereby allowing prompt treatment and input from specialist teams.

The precipitating factors should be addressed and monitored, which in this case were concurrent evidence of infection and poor medication compliance secondary to schizophrenia. Secondly, it is important to be aware that patients with mental health disorders are at risk of poor medication compliance and thus require more vigorous monitoring through regular blood tests and follow-up with a physician either in a primary care or secondary care setting to reduce the risk of such severe presentations. The use of directly observed therapy in certain conditions such as tuberculosis would have a potential role in patients that have poor medication compliance with other conditions predisposing to life-threatening events. This required administration of intravenous antibiotics and commencing a combination of thyroid hormone analogues. Additional measures involved in the management of myxedema coma include correction of electrolyte abnormalities and passive measures such as thermoregulation and blood glucose monitoring. The ongoing management and escalation of treatment in refractory cases depend on a careful assessment of clinical and biochemical parameters.

The use of liothyronine should only be prescribed in combination therapy with levothyroxine only if supervised and initiated by an Endocrinologist as per a joint British Thyroid Association (BTA) and Society for Endocrinology consensus statement. The use of such a combination therapy would be indicated in circumstances such as in overt hypothyroidism with a TSH level >10 mU/L, but also in patients with persistent symptoms of primary hypothyroidism whilst on levothyroxine providing other causes have been excluded and both adequate dosage and duration of levothyroxine monotherapy are ensured [11,12]. Upon commencement of combination therapy, BTA recommends dose adjustment of levothyroxine by lowering it to one-third that of the actual dose patient was taking and the dose of liothyronine being at approximately 1:17 of the current pre-combination levothyroxine dosage. Patients should be monitored for side effects and efficacy of combined therapy by assessing TSH response [11]. The use of combination therapy is recommended to be continued for up to 3 to 6 months before reviewing efficacy in those patients who were symptomatic on levothyroxine monotherapy. Liothyronine should not be prescribed as a monotherapy in hypothyroidism, including in cases of pregnancy. In addition to the above, it is recommended that once patients feel well on combined therapy and TSH remains stable within normal range, a discussion should take place with an Endocrinologist to assess the benefit of continued combined therapy and consideration of a trial of levothyroxine as a monotherapy again [11].

Elkattawy et al reported a case of myxedema in a patient with a long lie at home and altered mental status who was hypothermic, and bradycardic requiring ICU admission. The patient had an elevated creatine kinase (CK) level of 20,000 units/L (55-170 units/L), creatinine of 1.52 mg/dl (0.7-1.2 mg/dL), thyroid-stimulating hormone (TSH) of 97.62 mIU/L (0.4-4 mIU/L) and free T4 of 0.00 ng/dL (0.9-2.3 ng/dL). She responded well to treatment and made a complete recovery and her levothyroxine dose was increased following an endocrinology review [5]. These patients usually require long-term support which can be challenging in the community settings. Studies have shown that motivational interviewing, daily medication reminders to patients at home, and education sessions focused on disease management, medication compliance, and relapse were beneficial to patients' adherence [13,14]. The two interventions with the maximum effect were combining motivational interviewing techniques with patient-tailored education [14,15].

## Conclusions

Suspecting myxedema coma can be a challenging task, especially in patients presenting with sepsis and multiple electrolyte abnormalities who are haemodynamically unstable. Patients with known hypothyroidism and poor compliance with the treatment secondary to mental health issues warrant listing thyroid dysfunction as contributing factors to delayed response to treatment and slow recovery especially from ailments such as sepsis. Annual or bi-annual review of thyroid function is of grave importance as the chronic nature of conditions associated with the thyroid gland could easily lead to fading of compliance. Establishing a good clinician-patient relationship is key to improving medication compliance and care of psychiatric illness in patients with mental health issues. Our patient's past medical history of mental health issues was another factor that supported a more vigilant approach toward monitoring and dose adjustment of levothyroxine which otherwise would have prevented the current presentation.
